# MiR-124-3p negatively impacts embryo implantation via suppressing uterine receptivity formation and embryo development

**DOI:** 10.1186/s12958-024-01187-w

**Published:** 2024-01-31

**Authors:** Kezhen Yao, Quanmin Kang, Kai Chen, Biwei Shi, Xiaofen Jin

**Affiliations:** 1 Department of Reproductive Endocrinology, Women’s Hospital, Zhejiang University School of Medicine, No. 1 Xueshi Road, Hangzhou, China; 2 Department of Reproductive Endocrinology, Key Laboratory of Reproductive Genetics, Ministry of Education, Women’s Hospital, School of Medicine, Zhejiang University, Hangzhou, China; 3 China United Engineering Corporation Limited, Hangzhou, China; 4 Zhejiang Provincial Key Laboratory of Precision Diagnosis and Therapy for Major Gynecological Diseases, Women’s Hospital, Zhejiang University School of Medicine, Hangzhou, Zhejiang China

**Keywords:** miR-124-3p, Uterine receptivity, Embryo development, Re-methylation, Peri-implantation

## Abstract

During embryo implantation, blastocyst interacts with the receptivity endometrium and the endometrial epithelium secretes nurturing fluid to support embryonic development. Interferon-λ (IFN-λ) is a novel, non-redundant regulator that participates in the fetal–maternal interaction; however, the precise molecular mechanism underlying its impact on uterine receptivity remains elusive. Here, microarray profiling revealed that 149 specific miRNAs were differentially expressed in the human endometrial cells following IFN-λ treatment. In particular, miR-124-3p expression was significantly reduced after IFN-λ treatment (*p* < 0.05). An in vivo mouse pregnancy model showed that miR-124-3p overexpression notably decreased embryo implantation rate and led to an aberrant epithelial phenotype. Furthermore, miR-124-3p negatively impacted the migration and proliferation of endometrial cells, and hindered embryonic developmental competence in terms of blastocyst formation and global DNA re-methylation. Downstream analysis showed that *LIF*, *MUC1* and *BCL2* are potential target genes for miR-124-3p, which was confirmed using western blotting and immunofluorescence assays. In conclusion, IFN-λ-driven downregulation of miR-124-3p during embryo implantation modulates uterine receptivity. The dual functional role of miR-124-3p suggests a cross-talk model wherein, maternal endometrial miRNA acts as a transcriptomic modifier of the peri-implantation endometrium and embryo development.

**Supplementary Information:**

The online version contains supplementary material available at 10.1186/s12958-024-01187-w.

## Introduction

The establishment of a receptive endometrium is crucial for successful embryo implantation and pregnancy initiation. Appropriate progression of pregnancy necessitates the implantation of a competent blastocyst into a receptive endometrium. Specifically, reproductive failure is often attributed to inadequate endometrial receptivity [7], embryo quality, immunological factors and endometrial microenvironment [17]. The endometrium of the uterus is only receptive to the blastocyst during a brief period referred to as the "window of implantation" (WOI). During this period, the endometrium undergoes morphological and molecular changes that facilitate embryonic implantation, including the loss of epithelial cell polarity, formation of pinopodes, and altered profile of adhesive molecules and cytokines [22]. Despite huge progress made in the understanding of reproductive processes, the mechanisms underlying post-transcriptional regulation and implantation failure remain elusive.

MicroRNAs (miRNAs) are endogenous, non-coding RNAs that play a crucial role in the post-transcriptional regulation of gene expression via selectively targeting specific mRNAs for degradation or translational repression [10]. Nearly all cell types are capable of secreting miRNAs [15], and miRNAs functions as pivotal regulators of gene expression. With this increased recognition of miRNAs, their active role in modulating embryonic development, endometrial physiology, and embryo-maternal communication has been extensively investigated in the context of the endometrium [20, 23, 27]. During the human menstrual cycle and embryo implantation, various types of miRNAs are expressed and secreted by the uterine endometrium that are associated with uterine receptivity and stromal decidualization. These miRNAs belong to the miR-23, miR-30, miR125, miR-200, and miR-183 families [1, 32, 40]. Furthermore, specific miRNAs highly expressed in human embryonic cells also regulate blastocyst activation, embryo implantation, and development [13, 26].

We previously reported the crucial regulatory roles of IFN-λ (also known as IL-28/29) in maintaining normal endometrial functions evidenced by abnormally low levels of endometrial IFN-λ expression in women experiencing recurrent implantation failure [38, 39], however, the exact basis for the molecular mechanism underlying the action of IFN-λ remains unknown. Therefore, in this study, we employed miRNA microarray to identify miRNAs specifically regulated by IFN-λ that are involved in the regulation of normal endometrium. We screened several miRNAs for potential differential expression in IFN-λ treated endometrial cells and found a subset of these miRNAs to be associated with molecular events occurring at the maternal–fetal interface. The expression profiling of miRNAs allowed us for the identification of corresponding specific genes that may be post-transcriptionally repressed in human endometrial cells.

Aberrant miRNA expression is implicated in the pathogenesis of human endometrial disorders, including endometriosis, carcinoma and sterility [8, 24, 33]. Previous studies have reported the aberrant upregulation of miR-124-3p in both endometrial and serum samples obtained from women diagnosed with endometritis [6]. Another study reported the specific detection of miR124-3p in the exosomes in uterine fluid and its associated mucus, as well as its transfer from endometrial epithelium into the uterine cavity [21]. However, its precise role in endometrial receptivity and its ultimate impact on embryo implantation remain elusive. Further studies on miR-124-3p are warranted to elucidate its contribution to the essential endometrial-embryo cross talk. In the present study, we aimed to investigate the impact of miR-124-3p overexpression on endometrial receptivity and embryo development.

Our findings suggested that miR-124-3p, reduced by IFN-λ, plays a negative role in embryo implantation by suppressing both endometrial receptivity formation and blastocyst development. Exploring the function of the miR-124-3p and its potential target genes in endometrial receptivity and embryo implantation can improve the collective understanding of the cellular functions and molecular pathways by IFN-λ during peri-implantation period. The aforementioned findings emphasize the significance of miRNA upon IFN-λ treatment in regulating uterine receptivity during embryo implantation, thereby highlighting their potential as a promising biomarker for clinical applications.

## Materials and methods

### Animal ethics and rights

Here, 4–6-week-old adult C57BL/6 J female and male mice (of the same age) were purchased from Vital River (China). The average weights of the mice (before the experiments were initiated) were 20.2 ± 1.9 g (male), and 16.8 ± 1.8 g (female). All animal experiments were performed in accordance with the Guide for the Care and Use of Laboratory Animals and were approved by the Institutional Animal Care and Use Committee of Zhejiang University.

The animals were housed under standard conditions of 24 ± 2 °C and 40–70% humidity, with a 14 h light/10 h dark cycle. Animals were kept in groups of ≤ 5, and fed a standard diet for three weeks. 

### Cell culture, RNA isolation, and small RNA sequencing

Ishikawa cells were seeded on 6-well plates, treated with IFN-λ (100 ng·ml^−1^) for 6 h, and used as controls. Three independent experiments were conducted to prepare samples for each treatment. Total RNA was extracted using TRIzol reagent (Life Technologies, Carlsbad, CA, USA) following the manufacturer’s instructions. RNA integrity was assessed using an Agilent Bioanalyzer 2100 system (Agilent Technologies, Santa Clara, CA, USA). The total RNA from each sample was used for small RNA-seq performed by Novogene Co. Ltd. (Tianjin, China) on an Illumina Nova 6000 sequencing system. Library preparation, sequencing, and quality and quantity control of raw sequences were performed as previously described [35]. Sequencing data were submitted to the NCBI SRA (PRJNA971763).

### Bioinformatics analysis

Read counts were normalized by using the reads per kilobase feature via the million mapped reads method. Following normalization, the fold-change between treatment and control sample was calculated according to Eq. (1):

1 $$fold-change\;(FC)=log2\;(treatment/control)$$

and the *p*-value was calculated using the previously described formula [35]. The criteria used to select the differentially expressed genes were: |log2FoldChange|> 0.5 and *p*-value < 0.05. Gene Ontology (GO) and KEGG pathway analyses were performed using R program. Bioinformatics online software TargetScan (http://www.targetscan.org, Whitehead Institute, Cambridge, MI, USA) was used to predict whether a seed region of hsa-miR-124-3p existed within the 3’-UTR of human genes.

### Validation of differentially expressed miRNAs by quantitative PCR (qPCR)

MiRNA expression was validated using poly (A)-tailed quantitative PCR (qPCR). Total RNA was extracted from macrophages using Trizol reagent, and then 2 μg total RNA was reverse-transcribed using miScript II RT Kit (Qiagen GmbH, Hilden, Germany) according to the manufacturer’s instructions. qPCR was performed using SYBR Premix Ex Taq II (Tli RNase H Plus; Takara, Dalian, China) on a StepOnePlus PCR system (Applied Biosystems, Foster City, CA). All primers used are listed in Table S1a.

### Generation of adeno-associated virus-expressing miR-124-3p

Adeno-associated virus (AAV)-expressing mmu-miR-124-3p or mmu-miR-control cells were obtained from HANBIO (Shanghai, China). mmu-miR-124-3p was cloned downstream of the mouse CMV promoter into an AAV vector. Vector genomes with AAV inverted terminal repeat sequences were cross-packaged into AAV capsids via triple transfection of AAV-HEK293 cells. Infectious particles were purified by ammonium sulfate fractionation and iodixanol gradient centrifugation.

### Animal model and mmu-miR-124-3p-AAV injection

Mice were anesthetized with 30 mg·kg^−1^ ketamine, and 10 μL mmu-miR-124-3p-AAV (5 × 10^11^ virus particles) was injected into the bilateral uterine horn at a rate of 2 μL·min^−1^ via a micro-injection pump. A similiar volume of mmu-miR-control-AAV was injected into the control group. After 72 h, fertile male and AAV-injected female mice were reared together in cages. Day 1 of pregnancy (D1) was validated based on the presence of a vaginal plug the following morning. On day 5 of pregnancy, implantation sites were visualized following tail vein injection of trypan blue dye solution before the mice were sacrificed, and the uteri were excised. The number of implantation sites was recorded.

### Transfection of mimics, inhibitor of hsa-miR-124-3p in Ishikawa cells

Ishikawa cells growing in logarithmic phase were seeded into 6-well plates (10^5^ cells per·well), and maintained in RPMI-1640 medium supplemented with 10% FBS at 37°C in 5% CO2 for 24 h. The hsa-miR-124-3p mimic (5’-UAAGGCACGCGGUGAAUGCC-3’, Cat.No.miR1180418101457-1–5; RiboBio, China), hsa-miR-124-3p inhibitor (5’-GGCAUUCACCGCGUGCCUUA-3’, Cat.No.miR2180511012627-1–5, RiboBio), negative control mimic (Cat.No.miR1N0000001-1–5, RiboBio), and the negative control inhibitor (Cat.No.miR2N0000001-1–5, RiboBio) were transiently transfected into 80%–90% confluent Ishikawa cells using Lipofectamine 3000 transfection reagent (L3000015, Thermo Fisher Scientific, USA). All procedures were performed according to the manufacturer’s instructions.

### Migration assay

Ishikawa cells were grown in 60 mm plate and subjected to transfection. Subsequently, cells were wounded by removing a 500 to 700 µm wide strip with a standard 200 µL pipette tip and photographed, and designated as time 0 h. The cells were then incubated for 24 h before being imaged using the same procedure. The migration distance was then calculated using ImageJ software (*v.*1.5).

### Apoptosis assays

Transfected Ishikawa cells were seeded in 12-well plates and cultured for 24 h. Apoptosis was determined by staining with Alexa Fluor 488-conjugated Annexin V and propidium iodide (PI) (Molecular Probes, Eugene, OR, USA), and analyzed via flow cytometry (BD Biosciences, San Jose, CA, USA) to sort viable cells. Cells were further gated based on forward versus side scatter, and then analyzed after staining with a conjugated dye.

### Cell proliferation

Transfected Ishikawa cells were seeded in 96-well plates and cultured for 0, 12, 24, and 48 h. Cell proliferation was determined using Cell Counting Kit-8 (CCK-8; Beyotime, Jiangsu, China). The OD450 values of different cells were measured using a microplate reader (DNM-9602, Pulangxin, China).

### Preparation of IVF mouse embryos and transduction of embryos with mmu-miR-124-3p-AAV

IVF embryos were prepared as previously described [11]. In brief, superovulation was induced in female mice via an injection of 5 IU of PMSG, followed by injecting 5 IU hCG 48 h later. The cumulus-oocyte complexes (COCs) in the oviducts were collected after an additional 16 h, and transferred to HTF medium. To prepare sperm, the cauda epididymides were removed from mature male mice. The excised specimens were compressed to release a dense mass of spermatozoa, from which drops (~ 2 µL) were collected and placed in a dish containing 0.4 mL HTF medium, and cultured at 37°C in a CO_2_ incubator for 45 min. A total of 5–10 µL of sperm suspension were collected and introduced into each drop containing the COCs. After insemination for 6 h, the zygotes were washed several times with KSOM. The washed zygotes were cultured in 20 μL KSOM at 37 °C with 5% CO_2_ until use. Transduction of embryos with miR-124-3p was performed as previously described [25], where 1 μL mmu-miR-124-3p-AAV (5 × 10^10^ viral particles) was added to a drop of KSOM seeded with two-celled mouse embryos.

### Immunofluorescence and confocal microscopy analysis

Mouse embryos were fixed with 3.7% formaldehyde in phosphate-buffered saline (PBS) for 30 min, incubated with DAPI (Abcam, Cat. ab104139, Cambridge, UK) to visualize the nuclei, washed with PBS, and mounted onto microscope slides. The inner cell mass (ICM) and DNA methylation were stained with anti-Nanog and anti-5mC or Dnmt1 antibodies (Catalog numbers of primary antibodies used are listed in Table S1b) diluted in blocking solution. Following overnight incubation at 4°C, the cells were incubated with CoraLite488-conjugated goat anti rabbit-IgG and CoraLite594-conjugated goat anti mouse-IgG antibodies, and diluted in blocking solution for 2 h. After washing twice with PBS, the slides were examined, and images were acquired using a Leica STELLARIS 5 confocal microscope.

### Western blot analysis

Western blot was performed as described previously [35]. Catalog numbers of primary antibodies used are listed in Table S1b. The full uncropped blots images are showed in Figure S1.

### Statistical analysis

Data were averaged from ≥ 3 three independent experiments. All experimental data are represented as the mean ± SD, and were analyzed using either a Student’s *t*-test or one-way analysis of variance (ANOVA) with Duncan’s multiple range test in SPSS *v*.20 (IBM Corporation, Armonk, NY, USA). Statistical significance was set at *p* < 0.05. Differences are shown with *, **, ***, or different letters a, b, c and d.

## Results

### IFN-λ regulates miRNA expression in endometrial cells

**Fig. 1:**
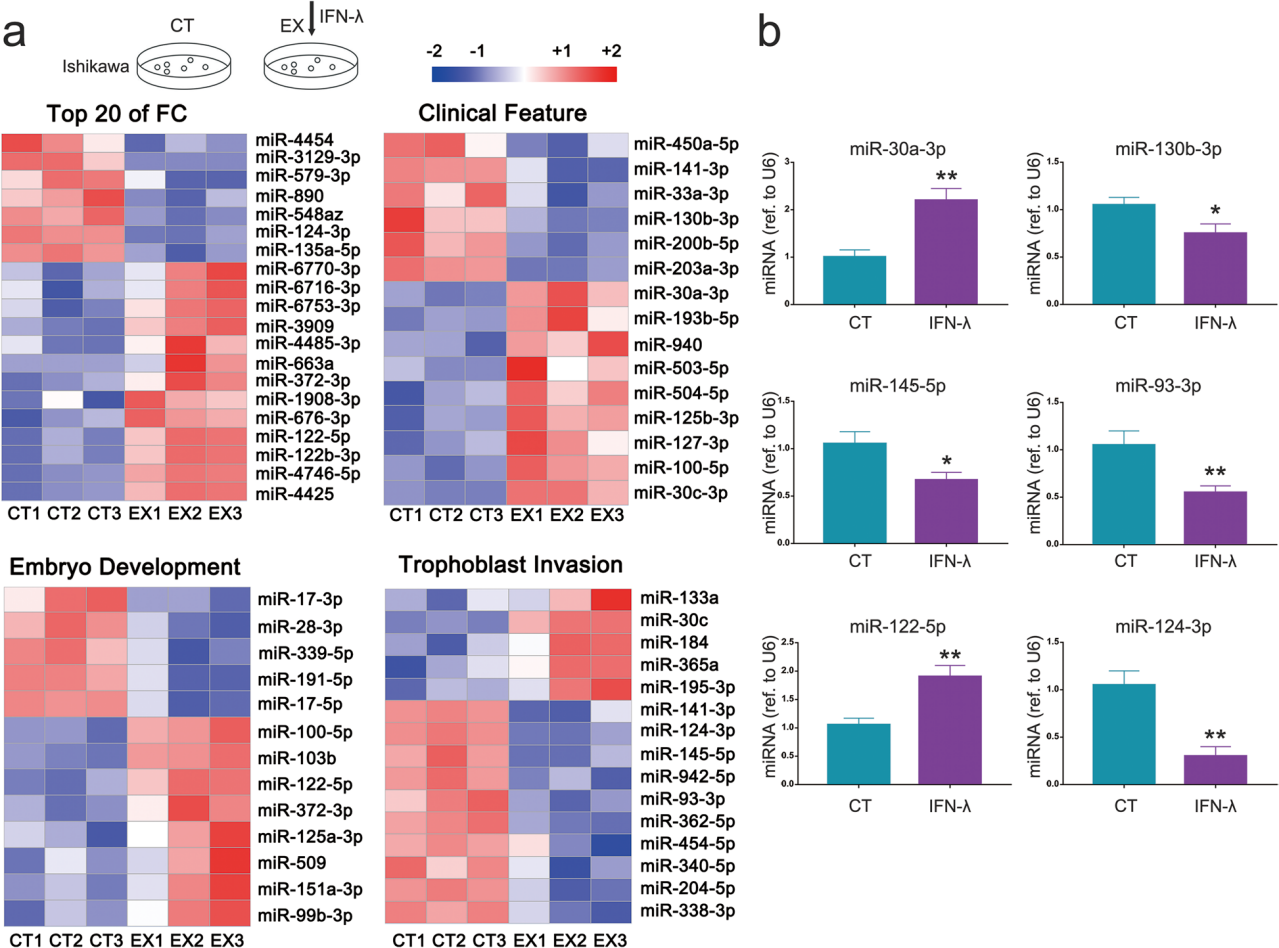
IFN-λ regulates miRNA expression in human endometrial cells. **a** Experimental scheme used for studying the miRNA expression profiles of Ishikawa cells treated with IFN-λ. Differentially expressed miRNAs were screened using a heat map according to the groups indicated. Blue and red indicate low and high expression, respectively. **b** Expression of each miRNA was determined using qPCR. IFN-λ enhances miR-30a-3p and miR-122-5p expression, and inhibits miR-130b-3p, miR-145-5p, miR-93-3p, and miR-124-3p expression. Data represent the mean ± SD of three independent experiments, *P < 0.05; **P < 0.01 (Student’s *t*-test)

To investigate whether miRNAs are involved in IFN-λ-mediated endometrial cell receptivity, small RNA sequencing was performed to identify IFN-λ-regulated miRNAs (Fig. 1a). It was found that IFN-λ affected the expression of 149 miRNAs in the Ishikawa cells, of which 65 were notably upregulated, and 84 were downregulated (*p* < 0.05, |log2FoldChange|) > 0.5) (Table S2). Top 20 miRNAs showing the highest fold change upon IFN-λ treatment are listed in Fig. 1a. The cluster analysis of literature search data on IFN-λ affected miRNAs (Table S3), revealed that 15 of those miRNAs have been reported to be associated with clinical infertility cases during the window of implantation. In addition, several miRNAs are linked to various biological processes during embryo–maternal interactions, including embryo development and trophoblast invasion (Fig. 1a). Considering the significance of these miRNAs involved in the maternal–fetal interface, the results suggest that IFN-λ-mediated endometrial cell to uterine receptivity by regulating miRNA expression.

qPCR analysis was performed to verify the small RNA sequencing data (Fig. 1b). Previous studies have reported that the upregulation of miR-30a-3p [19], or downregulation of miR-130b-3p [2], miR-145-5p [12] and miR-93 [9] enhances the endometrial cell receptivity. The data from the present study showed that miR-30a-3p and miR-122-5p were upregulated, while miR-124-3p, miR-130b-3p, miR-145-5p, and miR-93-3p were downregulated by IFN-λ treatment conditions in Ishikawa cells. Notably, miR124-3 and miR-122-5p were also among the top 20 differentially expressed miRNAs upon IFN-λ treatment in Ishikawa cells (|log2FoldChange|> 1.6, *p* < 0.01).

### Overexpression of miR-124-3p attenuated uterine receptivity and embryo implantation in a mouse model

**Fig. 2:**
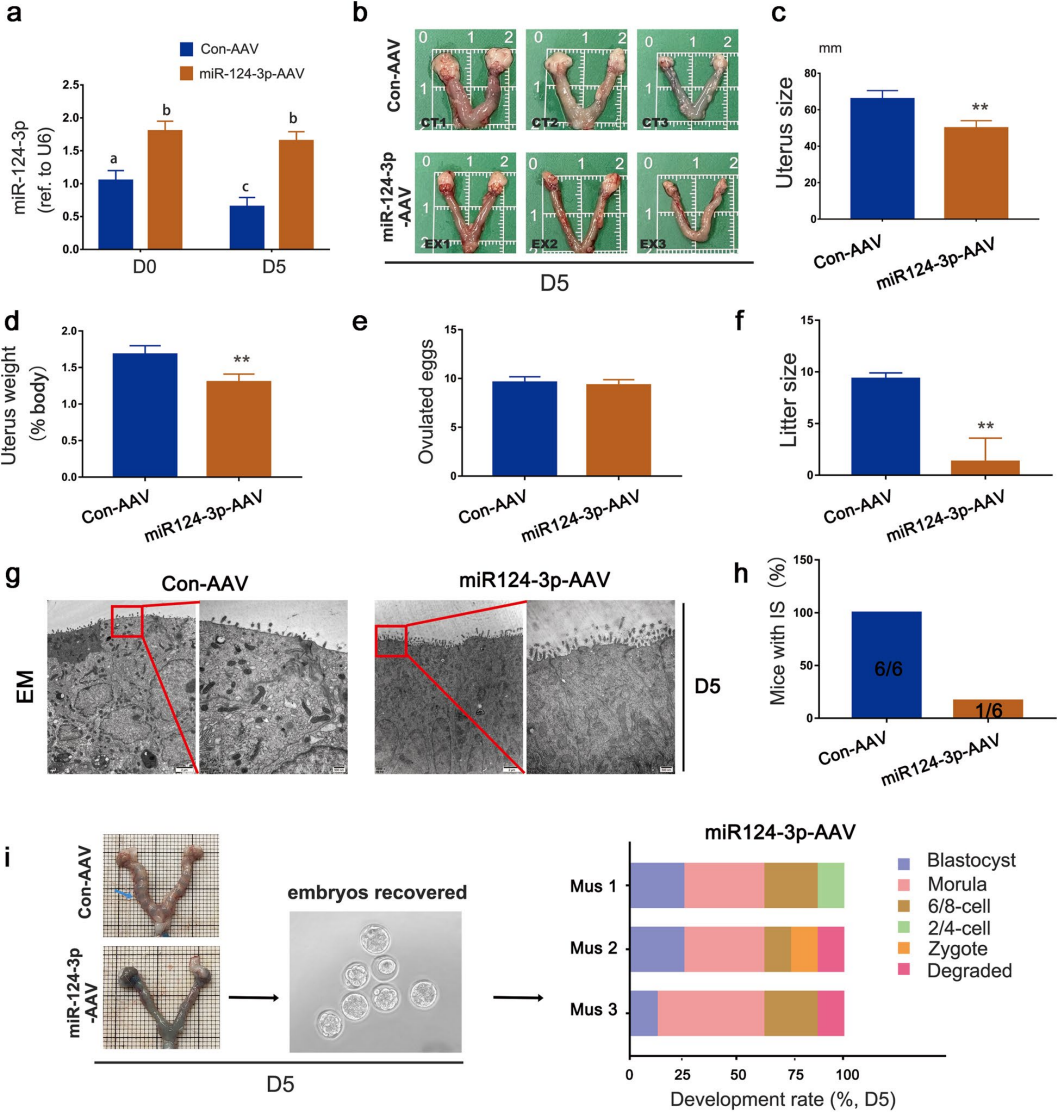
miR-124-3p-AAV delivery results in embryo implantation failure. **a** Delivery of miR-124-3p to mouse uteri of control and miR-124-3p-AAV groups was confirmed via real-time PCR analysis on days 0 and 5 of pregnancy. Data represent the mean ± SD of three independent experiments. Different superscripts indicate significance at *p* < 0.05 (one-way ANOVA with Duncan’s test). **b** Representative images showing uterus features of control and miR-124-3p-AAV groups crossed with wild-type males on day 5 of pregnancy. **c**, **d** Maternal uterine size and weight in the control and miR-124-3p-AAV groups on day 5 of pregnancy. Size was determined by measuring the length and diameter of the unilateral uterus (mm). Error bars represent the mean ± SD in replicates: ∗  ∗ *p* < 0.01 (Student’s *t*-test). **e**, **f** Number of ovulated eggs and average litter size in the control and miR-124-3p-AAV groups. Error bars represent the mean ± SD in replicates: ∗  ∗ *p* < 0.01 (Student’s *t*-test). **g** Electron microscopy (EM) analysis of mouse uterine epithelial surface showing impaired epithelial membrane transformation in the miR-124-3p-AAV delivery group. **h**, **i** Values within bars indicate the number of mice with implantation sites (ISs) per total number of tested mice. Blue arrow indicates the IS. A large proportion of mice in the miR-124-3p-AAV delivery group exhibited implantation failure, and embryos were easily recovered upon flushing the uterine horn on day 5 of pregnancy, without any signs of attachment. Bar charts show the embryo development rate in the miR-124-3p-AAV group on day 5 of pregnancy in vivo

To verify the role of miR-124-3p in female fertility, the uterine horn of non-pregnant female mice were injected with mmu-miR-124-3p-AAV or mmu-miR-control-AAV. These females were mated with fertile wild-type males, and the overexpression levels of miR-124-3p in the mice uteri were examined on the Day 5 of pregnancy (Fig. 2a). The size and weight ratio of uteri of pregnant mouse in miR-124-3p overexpressed group significantly decreased compared with that of control group (Fig. 2b, c, d). A normal number of ovulated eggs was noted in both overexpressed and control mice (Fig. 2e); however, the litter size was markedly lower in the miR-124-3p overexpressed females compared with the control females (Fig. 2f), suggesting that uterine miR-124-3p is crucial for normal female fertility.

Impaired epithelial membrane transformation exhibiting sustained long microvilli was observed in overexpressed miR-124-3p mice on day 5 of pregnancy (Fig. 2g). This aberrant epithelial morphology clearly indicated that defective uterine receptivity was induced by miR-124-3p. To identify the stage-specific failure of pregnancy in miR-124-3p overexpressed females, the implantation status was analyzed in these females. The results revealed that a large proportion (> 80%) of females exhibited implantation failure on day 5 of pregnancy (Fig. 2h) and embryos could be recovered by flushing the uterus without any signs of an attachment reaction. Notably, the stasis phenomenon of blastocyst formation was observed in some of the recovered embryos (Fig. 2i). These results clearly indicate that uterine miR-124-3p disrupts normal embryo implantation.

### MiR-124-3p plays a role in regulation of endometrial cell migration, proliferation, and apoptosis

**Fig. 3:**
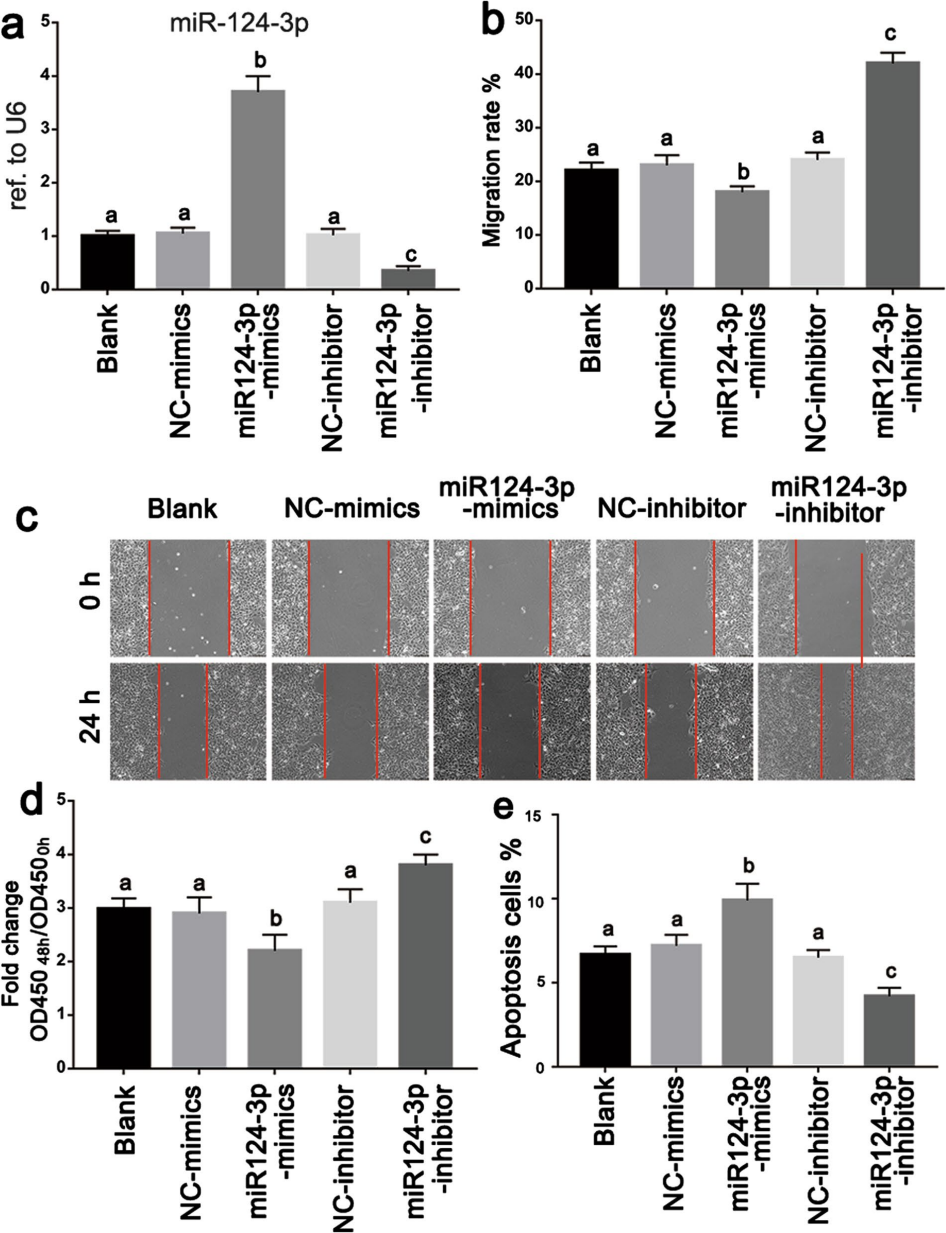
miR-124-3p inhibits migration and proliferation, inducing apoptosis on uterine epithelial cells in vitro. **a** Real-time PCR analysis for miR-124-3p expression in Ishikawa cells after miR-124-3p transfection with mimics and inhibitor. **b**, **c** Phase-contrast images of wound-healing assays to test the effect of miR-124-3p on cell migration. Data are presented as the size of surface area of Ishikawa cells entering the migration zone. **d** CCK-8 assay was used to evaluate cell proliferation capabilities of Ishikawa cells after transfection with miR-124-3p mimics and inhibitor. **e** Proportion of apoptotic cells was evaluated by Annexin V staining followed by flow cytometry analysis after miR-124-3p mimics and inhibitor transfection. All data represent the mean ± SD of three independent experiments. Different superscripts indicate significance at *p* < 0.05 (one-way ANOVA with Duncan’s test)

To further evaluate the function of miR-124-3p in human endometrium, a gain-of-function assay was performed by transfecting Ishikawa cells with a miR-124-3p mimic, miR-124-3p inhibitor, and the corresponding negative control (NC). The transfection efficiency was evaluated via qPCR (Fig. 3a). Treatment of Ishikawa cells with miR-124-3p mimic significantly attenuated the effect on migration; whereas treatment of Ishikawa cells with miR-124-3p inhibitor significantly promoted migration (Fig. 3b, c). Next, the CCK-8 assay was performed to determine the role of miR-124-3p in cell proliferation, and the results suggested that miR-124-3p overexpression inhibited Ishikawa cell proliferation (Fig. 3d). Moreover, apoptosis in miR-124-3p mimic-transfected cells was remarkably upregulated compared to that in mimic-NC cells (Fig. 3e). These results suggested that miR-124-3p may play an important role in the negative regulation of migration, proliferation, and anti-apoptotic capacity of endometrial cells.

### MiR-124-3p derails the developmental competence and global DNA methylation of embryos

**Fig. 4:**
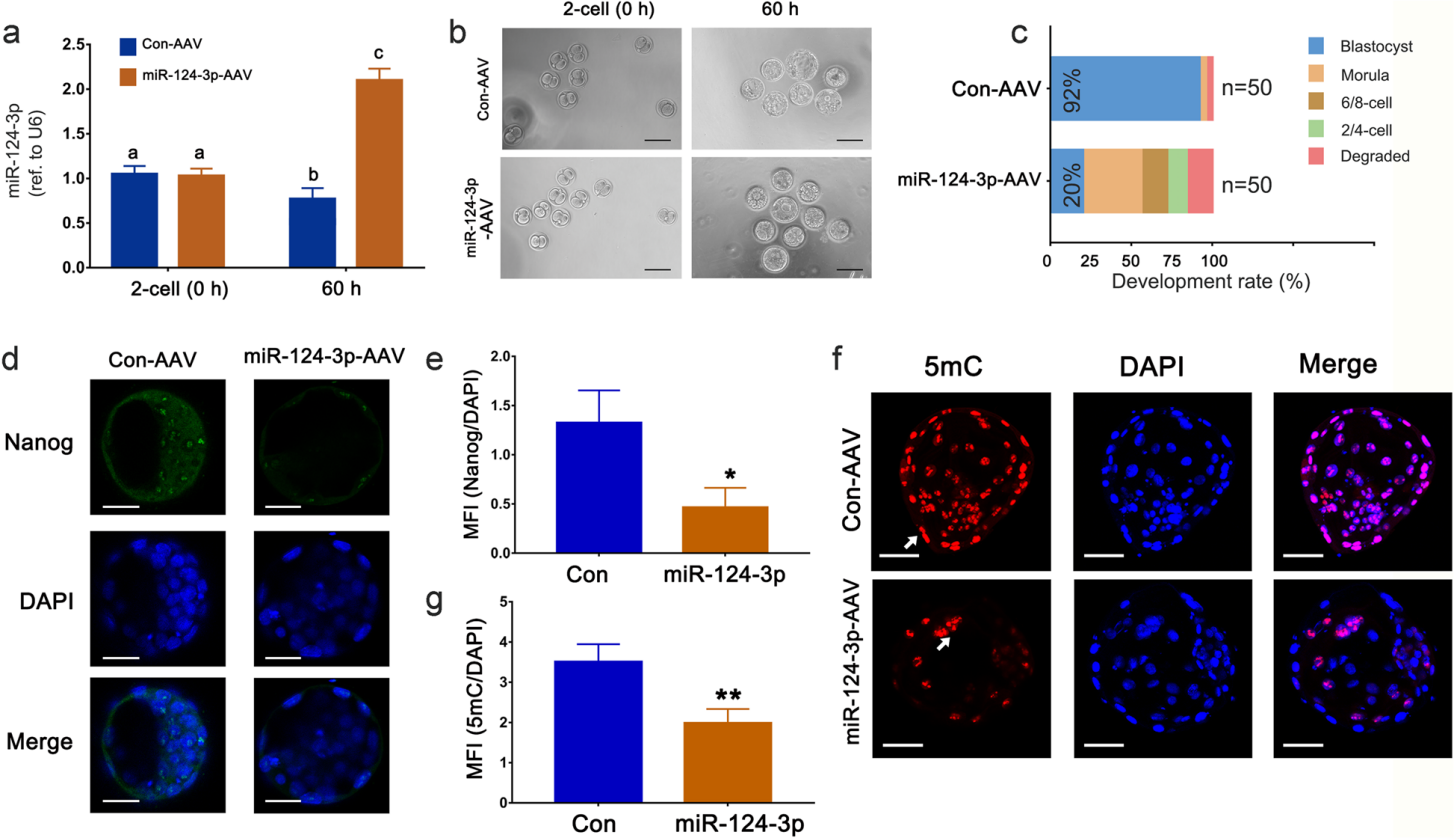
miR-124-3p derails blastocyst formation and global DNA methylation of embryos. **a** Real-time PCR analysis for miR-124-3p expression in control and miR-124-3p-AAV group embryos at the initiation (2-cells) and 60 h after transduction. Data represent the mean ± SD in replicates. Different superscripts indicate significance at *p* < 0.05 (one-way ANOVA with Duncan’s test). **b** Representative images of control and miR-124-3p-AAV group embryos at the initiation (2-cells) and 60 h after transduction. One representative image from six independent experiments is shown. Scale bar, 100 μm. **c** Bar charts showing the development rate of control and miR-124-3p-AAV group embryos 60 h after transduction. **d**, **e** Nanog staining (green signals) stands as an indicator of inner cell mass (ICM). Error bars represent the mean ± SD in replicates. ∗ *p* < 0.05 (Student’s* t*-test). **f**, **g** 5mC staining (red signals), as an indicator of global DNA methylation, was restricted to the nuclei of both trophoderm cells and ICM cells, demonstrating a severe reduction in the miR-124-3p-AAV group. Error bars represent the mean ± SD in replicates. **p < 0.01 (Student’s *t*-test). Scale bar: 20 µm

Maternal miRNAs may act as transcriptome modifiers in peri-implantation embryos [32]. The hindrance to blastocyst formation in miR-124-3p overexpressed females suggests a crucial role for miR-124-3p in embryonic development. The mmu-miR-124-3p-AAV and control AAV were added to the harvested two-celled mouse embryos and transduced for 60 h. The transduction efficiency of AAV in the embryos was validated using qPCR (Fig. 4a. The miR-124-3p overexpression group showed a remarkably reduced blastocyst formation rate compared to the control (92% vs. 20%; Fig. 4b, c). Furthermore, the percentage of ICM cells in the miR-124-3p overexpression group was significantly lower than that in the control group (Fig. 4d, e), indicating that miR-124-3p inhibited embryonic developmental potential. Next, global DNA methylation in embryos was assessed via 5mC staining and the results revealed that it was predominantly localized in cell nuclei and more abundant in trophectoderm cells than in ICM cells. Notably, a significant reduction of 5mC was observed in the miR-124-3p overexpression group compared to that in the control (*p* < 0.05) (Fig. 4f, g).

### Verification of potential target genes of *miR-124-3p* in endometrial cells and embryos

**Fig. 5:**
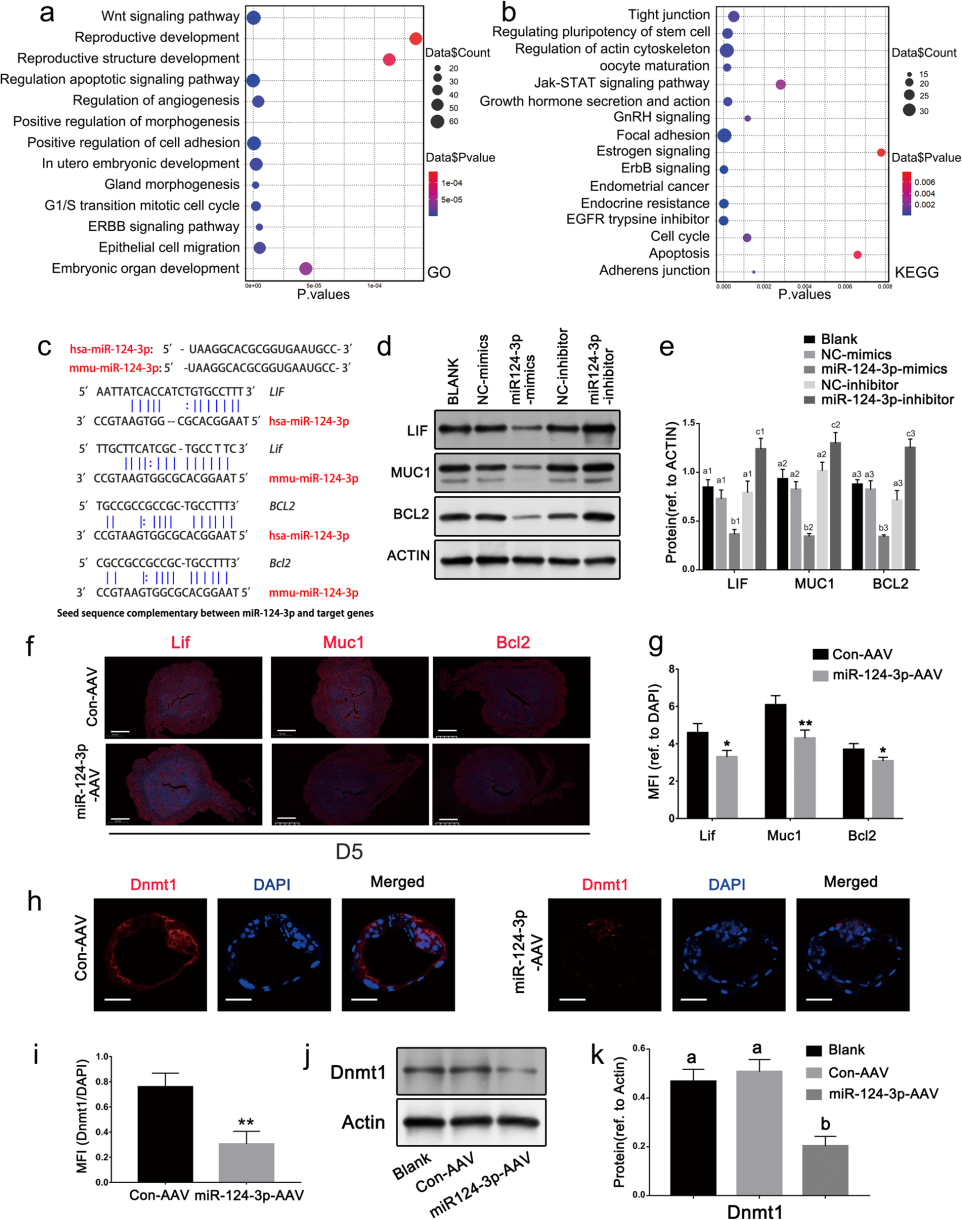
Preliminary identification of target genes of miR-124-3p in the endometrium and embryo. **a** Gene ontology (GO) annotation was performed for the target genes of miR-124-3p, and the terms according to the protein function are plotted. **b** KEGG pathway analysis of target genes of miR-124-3p. **c** Complementarity between target genes and seed sequence of miR-124-3p. **d**, **e** Western blot analyses show increased levels of LIF, MUC1, and BCL2 proteins in Ishikawa cells transfected with miR-124-3p inhibitor. Data represent the mean ± SD of three independent experiments. Different superscripts indicate significance at* p* < 0.05 (one-way ANOVA with Duncan’s test). **f**, **g** Immunofluorescence staining was performed to compare the expression levels of Lif, Muc1, and Bcl2 in the uteri from the control and miR-124-3p-AAV groups on day 5 of pregnancy. Scale bar: 500 μm. Histogram shows the ratio of integrated density level to the area. Data represent the mean ± SD in replicates. *p < 0.05; **p < 0.01 (Student’s *t*-test). **h**, **i** Dnmt1 localization in control and miR-124-3p-AAV-treated blastocytes following 2-cells formation for 60 h. Dnmt1 signals (red) localize to both TE and ICM cells. Severe inhibition of Dnmt1 was characterized by a lack of signal in the miR-124-3p-AAV group. Scale bar: 20 µm. **p < 0.01 (Student’s *t*-test). **j**, **k** Western blotting analysis of Dnmt1expression in blastocytes after control and miR-124-3p-AAV transduction. Actin was used as an internal control. Data represent the mean ± SD in replicates. *p* < 0.05 (one-way ANOVA with Duncan’s test)

All potential target genes of miR-124-3p probably target genes (validated beforehand) are listed in Table S4. Several of these genes such as *LIF*, *MUC1*, and *STAT3*, were previously shown to be regulated by IFN-λ [38, 39]. Numerous genes are involved in the implantation and early embryonic development, including *WNT4, BCL2, BAX, DNMT1, KLF4* and *TIMP3*. Gene Ontology (GO) annotation and KEGG pathway enrichment analyses were performed to classify proteins according to their associated biological processes and signaling pathways (Fig. 5a, b). Substantial enrichment was observed in terms of cell adhesion, cell migration, mitotic cell cycle, angiogenesis, progesterone–estrogen signaling, *Wnt* signaling, apoptotic signaling, and in utero embryonic development. In-silico analysis was conducted to assess the complementarity between target genes and the seed sequence of miR-124-3p in both human and mouse system, revealing conserved similarities across human and mouse species (Fig. 5c). These findings suggest that miR-124-3p plays an important role in the morphogenesis of endometrial receptivity and embryonic development at the maternal–fetal interface by regulating the expression of its target genes.

The regulatory function of miR-124-3p was further confirmed by modulating the expression of *LIF*, *MUC1,* and *BCL2* expression levels. Western blot analysis was of Ishikawa cells transfected with either the miR-124-3p mimic or inhibitor, showed that LIF, MUC1, and BCL2 protein levels were downregulated and upregulated, respectively (Fig. 5d, e). The expression levels of these proteins in the uteri of miR-124-3p overexpressed mice were detected by immunofluorescence, and the results were consistent with those observed in Ishikawa cells (Fig. 5f, g).

The protein level of Dnmt1 on mouse embryonic day 5 was determined by immunofluorescence staining. Here, Dnmt1 expression in embryos transfected with miR-124-3p-AAV was notably lower than that in embryos transfected with NC-AAV (Fig. 5h, i). A similar result was obtained using western blotting (Fig. 5j, k).

## Discussion

Implantation is a complex process that necessitates the synchronized development of a viable embryo and receptive endometrium. Multiple molecules, including hormones, cytokines, chemokines, immune factors and receptors participate in the regulation of implantation via autocrine, paracrine, and juxtacrine mechanisms [41]. Aberrant expression of critical genes in the endometrium during the implantation window has a negative impact on embryo implantation, and miRNA expression plays a pivotal role in regulating this process as well as determining subsequent pregnancy outcomes. We previously identified IFN-λ as a novel, non-redundant regulator that participates in the fetal–maternal immune interaction [38, 39]. In the present study, microarray profiling revealed 149 differentially expressed miRNAs in human endometrial cells following IFN-λ treatment. Researchers have identified several miRNAs that are differentially expressed during the WOI, or are involved in various biological processes in embryo–maternal interactions, including embryo development and trophoblast invasion, thereby indicating that these IFN-λ-mediated miRNAs may play a pivotal role in embryo implantation.

Many miRNAs regulated by IFN-λ play regulatory roles in endometrial receptivity. For example, the downregulation of miR-200 results in increased PTEN expression, which affects cell proliferation and apoptosis during decidualization [28]. MiR-503 is upregulated, while miR-203 is downregulated in the mid-secretory endometrium [16]. Additionally, miR-30 and miR-940 are upregulated,whereas miR-450 is downregulated in the receptive endometrium compared with the pre-receptive endometrium [32, 37]. Repression of miR-181 expression on day 4 of pregnancy is crucial for the initiation of implantation because it downregulates *Lif*, a promising marker of implantation [4]. These findings suggest that IFN-λ modulates miRNA expression in endometrial cells, thereby steering endometrial maturation toward a receptive state,however, the precise functional significance of several strongly up- or downregulated miRNAs by IFN-λ remains elusive. Our data suggest that these candidate miRNAs may have significant implications for the initiation and maintenance of the receptivity window, as well as subsequent embryo implantation.

The expression of miR-124-3p has been reported to be upregulated in both endometrium and serum of women with chronic endometritis [6], whereas another study demonstrated its downregulation in endometrial cancers [3], thereby suggesting a regulatory role of this miRNA in endometrial cell proliferation, and that its altered expression may be associated with endometrial dysfunction. Here, an in vivo mouse pregnancy model showed that the overexpression of intrauterine miR-124-3p resulted in an aberrant epithelial phenotype and low embryo implantation rates. It was found that miR-124-3p exerted a negative effect on cell migration and proliferation, while the overexpression of miR-124-3p induced apoptosis in endometrial cells. The target genes validated for miR-124-3p are implicated in the mitotic cell cycle, cell migration and cell adhesion, and have been reported to be upregulated in the secretory phase of the female reproductive cycle. For example, *LIF* is expressed in the endometrial epithelium during the luteal phase of the menstrual cycle, which coincides with implantation time. Accordingly, impairments in endometrial LIF expression may significantly contribute to pathological processes involving implantation and infertility [14]. Mucin 1 is an integral transmembrane mucin glycoprotein expressed on the apical surface of endometrial epithelia. Furthermore, decreased MUC1 expression in endometrium is an independent receptivity marker in recurrent implantation failure during the WOI [34]. Decreased expression of the anti-apoptotic factor Bcl2 promotes apoptosis, in addition to affecting the proliferation and migration of stromal and decidual cells [18]. In the present study, miR-124-3p was confirmed to repress the expression of *LIF*, *MUC1*, and *BCL2*; therefore, we propose that the downregulation of miR-124-3p may positively regulate these receptivity biomarkers in terms of endometrial cell proliferation, migration, and apoptosis.

miRNAs encapsulated in exosomes/microvesicles can be transported to distant cells in tissues. Previous studies have demonstrated that maternally secreted miRNAs, which are transported via exosomes, can be internalized by blastocysts, subsequently altering the embryonic transcriptome and phenotype [32]. Notably, modified exosomes can efficiently deliver miR-124-3p between organs [36]. Validated target genes for miR-124-3p have also been implicated in utero embryonic organ development, suggesting that endometrial miR-124-3p may enhance the developmental capacity of peri-implantation embryos when transported via exosomes and modify the embryonic transcriptome and phenotype. Although a minimal level of methylation is observed during the blastocyst stage, the embryo undergoes genome-wide remethylation to create a canonical methylation landscape upon implantation [29]. The establishment of de novo methylation is likely attributed to the elevated levels of DNA methyltransferases (DNMTs) in embryos, while the two DNMT families (DNMT1 and DNMT3) are responsible for methylation establishment and maintenance [42]. Knockdown of DNMT1 expression disrupts peri-implantation embryo development [31]. The results from the present study indicated that the overexpression of miR-124-3p suppressed global DNA methylation and downregulated Dnmt1 expression in the blastocyst, suggesting that the abnormally low expression of IFN-λ in the endometrium may cause abnormal secretion of miR-124-3p during maternal–fetal interactions, thereby negatively impacting remethylation and the development of peri-implantation embryos. This evidence supports the notion that miRNAs differentially expressed in the maternal uterus during the WOI are transferred to offspring as epigenetic signals, and thus play various important roles in embryonic development.

The present study was subject to certain limitations. Further investigation is warranted to elucidate the underlying mechanism governing the translocation of miR-124-3p from the endometrium into the uterine cavity. miRNAs are secreted by cells and encapsulated within microvesicles, shielding them from RNase degradation and conferring upon them an extended half-life [30]. Notably, exosomes frequently serve as vehicles for the transport of miRNAs, playing a pivotal role in intercellular communication [5]. Previous studies have demonstrated the presence of miR-124-3p in exosomes present in uterine fluid and transfer from endometrial epithelium to the uterine cavity [21]. However, evidence supporting the involvement of miR-124-3p in embryo-uterus communication through exosomes remains inconclusive. We are actively addressing this question in our laboratory.

**Fig. 6:**
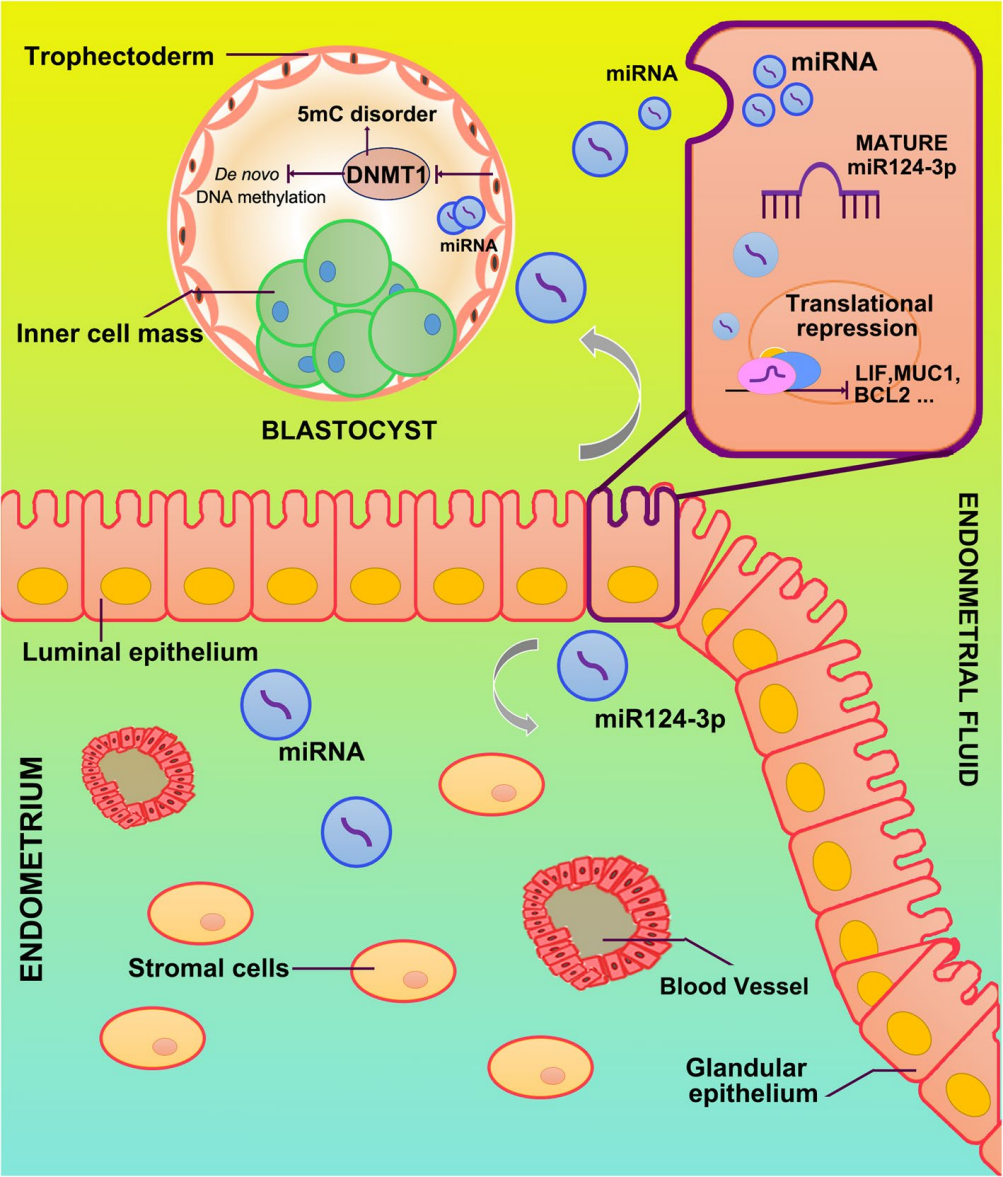
A hypothetical schematic depicting a novel mechanism of maternal–fetal cross-talk mediated by miR-124-3p. The aberrant expression of miR-124-3p, originating from the maternal endometrium, exerts a modulatory effect on both endometrial receptivity and embryonic epigenetics

In conclusion, this is the first study to report the pivotal role of miR-124-3p in regulating maternal–fetal interface expression, particularly that of receptivity biomarkers in the endometrium and embryo (Fig. 6). This regulation induces translational repression, which presents novel opportunities for improving implantation outcomes. Intra- or intercellular communication through IFN-λ-mediated miRNAs provide a novel perspective for understanding the mechanisms of implantation. In addition, IFN-λ-mediated miRNAs, especially extracellular miRNAs, may serve as non-invasive biomarkers in in vitro fertilization and embryo transfer for detecting and predicting embryo quality and endometrial receptivity; however, the package forms and secretion patterns of miR-124-3p remain elusive. Therefore, further studies are required to consider the potential impact of its function, as well as the accuracy of evaluation when utilizing miR-124-3p as a biomarker under specific conditions.

### Supplementary Information

**Additional file 1:** **Table S1a.** Primers for miRNAs. **Table S1b.** Antibody information.

**Additional file 2:** **Table S2.** Identification of differentially expressed miRNA in Ishikawa cells treated with IFN-λ compared to untreated cells.

**Additional file 3:** **Table S3.** Summary of previous studies of identified miRNAs expression/repression at the maternal-fetal interface.

**Additional file 4:** **Table S4a.** miR-124-3p target genes. **Table S4b.** Gene Ontology (GO) analysis. **Tabel S4c.** KEGG pathway analysis.

**Additional file 5:** **Figure S1.** The full uncropped blots images.

## Data Availability

The datasets generated or analyzed during this study are available from the corresponding author on reasonable request.
