# Supplementary material for: MiR-124-3p negatively impacts embryo implantation via suppressing uterine receptivity formation and embryo development
Source: Reprod Biol Endocrinol. 2024 Jan 31;22:16. doi: 10.1186/s12958-024-01187-w (PMC10829223; doi:10.1186/s12958-024-01187-w)
Supplement: Supplementary file 1 — Additional file 1: Table S1a. Primers for miRNAs. Table S1b. Antibody information. [file 12958_2024_1187_MOESM1_ESM.docx]

Table S1a Primers for miRNAs.

| Gene | Forward Primer Sequence (5’-3’) | Reverse Primer Sequence (5’-3’) |
| --- | --- | --- |
| hsa-miR-30a-3p | CCCTGCTCTGGCTGGTCAAACGGA | TTGCCAGCCCTGCTGTAGCTGGTTGAAG |
| hsa-miR-130b-3p | CTGGTAGGGTACAGTACTGTGATA | CTGGTGTCGTGGAGTCGGC |
| hsa-miR-145-5p | GUCCAGUUUUCCCAGGAAUCCCU | AGGGAUUCCUGGGAAAACUGGAC |
| hsa-miR-93-3p | ACACTCCAGCTGGGACTG | CTCAACTGGTGTCGTGGA |
| hsa-miR-124-3p | CGCGTAAGGCACGCGGTG | ATCCAGTGCAGGGTCCGAGG |
| hsa-miR-122-5p | GTGACAATGGTGGAATGTGG | CAGAACCGTAGCAAACGAAA |
| hsa-u6 | CTCGCTTCGGCAGCACAT | AACGCTTCACGAATTTGCGT |
| mmu-miR-124-3p | TGAGGGCCCCTCTGCGTGTTCA | TGAGGGCCCCTCTGCGTGTTCA |
| mmu-u6 | ATTGGAACGATACAGAGAAGATT | GGAACGCTTCACGAATTTG |

Table S1b Antibody information

| Antibodies | Company | Catalog/clone Number | Dilution | Application |
| --- | --- | --- | --- | --- |
| LIF (human/mouse) | Proteintech | 26757-1-AP | 1:200 | IF,WB |
| MUC1 (human/mouse) | Proteintech | 23614-1-AP | 1:200 | IF,WB |
| BCL2 (human/mouse) | Proteintech | 68103-1-Ig | 1:200 | IF,WB |
| Nanog (mouse) | Proteintech | 14295-1-AP | 1:200 | IF |
| 5mC (mouse) | Proteintech | 39649 | 1:200 | IF |
| Dnmt1 (mouse) | abcam | ab188453 | 1:200 | IF,WB |
| Actin | abcam | ab8224 | 1:500 | WB |
