# Supplementary material for: MiR-124-3p negatively impacts embryo implantation via suppressing uterine receptivity formation and embryo development
Source: Reprod Biol Endocrinol. 2024 Jan 31;22:16. doi: 10.1186/s12958-024-01187-w (PMC10829223; doi:10.1186/s12958-024-01187-w)
Supplement: Supplementary file 3 — Additional file 3: Table S3. Summary of previous studies of identified miRNAs expression/repression at the maternal-fetal interface. [file 12958_2024_1187_MOESM3_ESM.docx]

**Table S3** Summary of previous studies of identified miRNAs expression/repression at the maternal-fetal interface

|  | **miRNAs** | **Methods** | **PMID ID** |
| --- | --- | --- | --- |
| Clinical Feature | miR-450a-5p | endometrial epithelium/ microarray | PMID:26395145 |
|  | miR-141-3p | endometrial epithelium/ microarray | PMID: 26395145 |
|  | miR-33a-3p | placenta/ dual luciferase assay | PMID: 35325806 |
|  | miR130b-3p | endometrial samples/ qPCR | PMID: 28855728 |
|  | miR-200b-5p | endometrial fluid/ microarray | PMID: 36029522 |
|  | miR-203a-3p | myometrial tissue/ microarray | PMID: 36293200 |
|  | miR-30a-3p | endometrial epithelium/ microarray | PMID: 26395145 |
|  | miR-193b-5p | decidualized cells/ in-situ hybridization | PMID: 37657301 |
|  | miR-940 | endometrium/ microarray | PMID: 30231774 |
|  | miR-503-5p | endometrium/ microarray | PMID: 35563053 |
|  | miR-504-5p | endometrial samples/ microarray | PMID: 31796963 |
|  | miR-125b-3p | endometrium/ microarray | PMID: 35563053 |
|  | miR-127-3p | villus and decidua /qPCR | PMID: 29973278 |
|  | miR-100-5p | villus and decidua /qPCR | PMID: 29973278 |
|  | miR-30c-3p | endometrial samples/ robust rank aggregation | PMID: 28855728 |
| **Embryo Development** | miR-17-3p | oviductal fluids/ microarray | PMID: 35452772 |
|  | miR-28-3p | plasma/ qPCR | PMID: 36303988 |
|  | miR-339-5p | blastocoel fluid/ qPCR | PMID: 36081236 |
|  | miR-191-5p | sperm/ microarray;  blastocoel fluid/ qPCR | PMID: 32066367  PMID: 36081236 |
|  | miR-17-5p | oviductal fluids/ microarray | PMID: 35452772 |
|  | miR-100-5p | sEVs/ qPCR | PMID: 33376629 |
|  | miR-103b | oocytes and early embryos/ qPCR | PMID: 26062615 |
|  | miR-122-5p | blastocyst/microarray | PMID: 28421107 |
|  | miR-372-3p | culture media/ qPCR | PMID: 34256783 |
|  | miR-125a-3p | embryo/ qPCR | PMID: 27906131 |
|  | miR-509 | culture medium/ microarray | PMID: 36042522 |
|  | miR-151a-3p | placentas and plasma/ microarray; blastocysts/ qPCR | PMID: 24664294  PMID: 31199674 |
|  | miR-99b-3p | blastocysts/ qPCR | PMID: 28166979 |
| **Trophoblast Invasion** | miR-133a | placenta tissues/ qPCR | PMID: 31858525 |
|  | miR-30c | trophoblasts/ dual luciferase assay | PMID: 31926626 |
|  | miR-184 | trophoblasts/ qPCR | PMID: 30833572 |
|  | miR-365a | trophoblasts/ qPCR | PMID: 27577708 |
|  | miR-195-3p | trophoblasts/ qPCR | PMID: 35716001 |
|  | miR-141-3p | placenta tissues/ qPCR | PMID: 36427432 |
|  | miR-124-3p | trophoblast cell/ overexpress | PMID: 33010604 |
|  | miR-145-5p | trophoblast cell/ qPCR | PMID: 31669240 |
|  | miR-942-5p | placenta / qPCR | PMID: 33253995 |
|  | miR-93-3p | trophoblast cell/ qPCR | PMID: 32046397 |
|  | miR-362-5p | trophoblastic cells/ qPCR | PMID: 29665647 |
|  | miR-454-5p | trophoblastic cells/ qPCR | PMID: 33129036 |
|  | miR-340-5p | trophoblastic cells/ qPCR | PMID: 35809813 |
|  | miR-204-5p | Trophoblastic-like cells/ qPCR | PMID: 26003727 |
|  | miR-338-3 | trophoblast cell/ overexpress | PMID: 32056133 |
