# Supplementary figures and images for: MiR-124-3p negatively impacts embryo implantation via suppressing uterine receptivity formation and embryo development
Source: Reprod Biol Endocrinol. 2024 Jan 31;22:16. doi: 10.1186/s12958-024-01187-w (PMC10829223; doi:10.1186/s12958-024-01187-w)

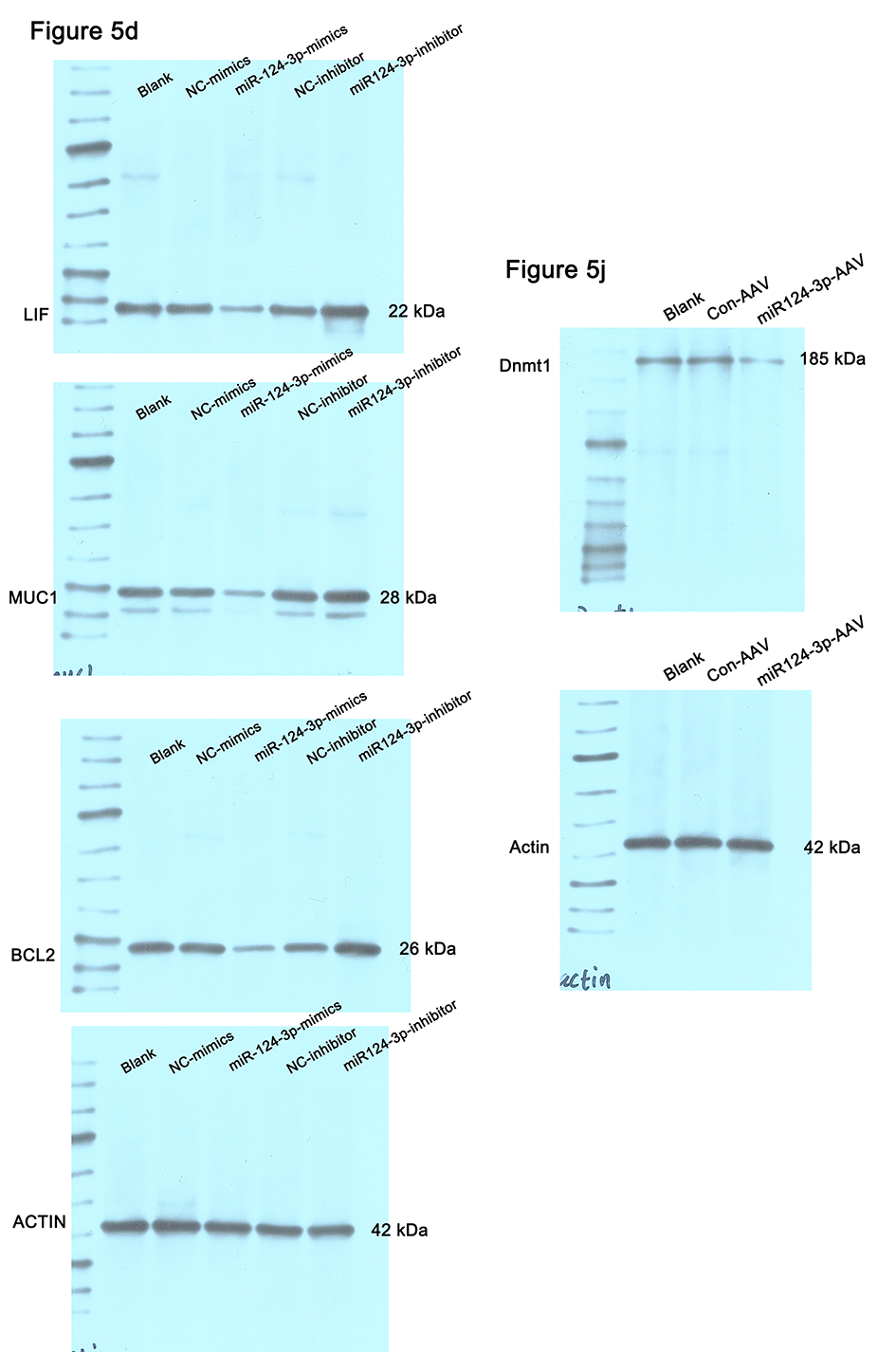

Supplement: Supplementary file 5 — Additional file 5: Figure S1. The full uncropped blots images. [file 12958_2024_1187_MOESM5_ESM.tif]
